# Root canal irrigants effect on the compressive strength and calcium ion release of Biodentine

**DOI:** 10.1016/j.heliyon.2024.e38267

**Published:** 2024-09-21

**Authors:** Devika Jain, Krishna Prasad Shetty, Alexander Maniangat Luke, Nidambur Vasudev Ballal

**Affiliations:** a Department of Conservative Dentistry and Endodontics, Manipal College of Dental Sciences, Manipal, Manipal Academy of Higher Education, Manipal, 576104, Karnataka, India; b Department of Clinical Science, College of Dentistry, Centre of Medical and Bio-Allied Health Science Research, Ajman University, Al-Jruf Ajman, P.O.BOX 346, United Arab Emirates

**Keywords:** Biodentine, Calcium ion release, Compressive strength, EDTA, Dual rinse HEDP, Sodium hypochlorite

## Abstract

Compressive strength and calcium ion release are integral properties of Biodentine for its enhanced efficiency. The present study evaluated the effects of Dual Rinse HEDP (DR HEDP), ethylenediaminetetraacetic acid (EDTA) and sodium hypochlorite (NaOCl) on the calcium ion release and compressive strength of Biodentine. Eighty Biodentine specimens were moulded and randomly divided into four groups (n = 20). Samples in group 1 were treated with 17 % EDTA; group 2 with DR HEDP; group 3 with 2.5 % NaOCl; and group 4 with distilled water. Samples were immersed in 10 mL of the test solutions for 1 min. The mean concentration of the calcium ion released was measured using atomic absorption spectrophotometry. The remaining 40 samples were tested for their compressive strength. Significant differences were determined among all the irrigants tested for calcium ion release and compressive strength. Samples treated with NaOCl had the lowest calcium ion release, while samples treated with 17 % EDTA had the largest calcium ions. No significant differences were measured between DR HEDP or distilled water. For compressive strength, samples treated with 2.5 % NaOCl had the lowest strength, while the highest values were obtained with distilled water. There was a significant difference between DR HEDP and EDTA, in which EDTA reduced the compressive strength significantly more than DR HEDP. DR HEDP had less detrimental effect on the calcium ion release and compressive strength of Biodentine.

## 1 Introduction

Apexification, vital pulp therapy, root-end filling, coronal seal, and perforation repair are the common endodontic procedures that use tricalcium silicate (TCS) based cement [1]. The cement is hydraulic in nature, which means that when moisture is present, it sets [2]. Mineral trioxide aggregate (MTA), Biodentine, and Bioaggregate all contain TCS as the primary cementitious component. When TCS-based materials contact synthetic tissue, they precipitate hydroxyapatite on their surface. The material's setting time is reduced, and compressive strength is increased by calcium carbonate and calcium sulfate [3,4]. TCS-based cements may come in direct contact with irrigation solutions during endodontic treatment. Some investigations have found that chelating agents have a negative impact on TCS containing products [[5], [6], [7]].

The TCS-based “Biodentine” material (Septodont, Saint Maur des Fosses, France) [8] was developed specifically as a “dentine replacement” substance and first became commercially available in 2009. Biodentine was developed to have faster setting and higher strength than other TCS type products. Biodentine releases calcium ions [9,10] but may be affected by irrigating solutions. Calcium ions stimulate mineralization-related signaling pathways [11,12].

An outstanding physical property of TCS cement is compressive strength (CS) [13,14]. In addition, in pulp therapies, the cement should have high compressive strength to resist functional and parafunctional stresses in the oral environment [15]. The compressive strength provides useful information on the setting of TCS cement. Several irrigating solutions are employed during endodontic therapy to disinfect the canal system and eliminate the smear layer [16]. Irrigants such as ethylenediaminetetraacetic acid (EDTA) and sodium hypochlorite (NaOCl) are commonly used [17,18]. NaOCl must be replenished during the procedure. 17 % EDTA is commonly used for eliminating the canal wall smear layer formed during instrumentation of a root canal system [19]. Previous research has demonstrated that EDTA in combination with NaOCl irrigation dramatically reduces the calcium to phosphorous ratio (Ca/P) of root canal dentin [[20], [21], [22]]. EDTA is a calcium-chelating agent that extracts calcium ions to dissolve minerals in the smear layer on the underlying dentine after prolonged contact [23]. Depending on the concentration of the solution, NaOCl may [22] or may not [20] alter the Ca/P ratio of superficial root canal dentin when administered alone.

Etidronic acid (HEDP or 1-hydroxyethane 1,1-diphosphonic acid) is a mild chelator used in endodontics. HEDP has no short-term reactivity with NaOCl and has been as a suggested replacement for EDTA or citric acid [24]. HEDP can be used in conjunction with a NaOCl solution, a technique known as “continuous chelation” [25]. The main clinical advantage of combining HEDP and NaOCl for root canal irrigation is ease and time saving. Dual Rinse HEDP (DR HEDP) (Medcem GmbH, Weinfelden, Switzerland) comes in the form of a capsule containing 0.9 g of etidronate powder, which should be blended with 10 mL of a NaOCl solution of choice (0.5–5.25 %) immediately before treatment, yielding an irrigant having both active chlorine and roughly 9 % HEDP [26]. It is as efficient as NaOCl in tissue dissolution [27] and is antibacterial and nontoxic and improves the bonding of sealers to root canal dentin [28].

No studies have evaluated the effectiveness of DR HEDP and EDTA on the CS and calcium ion release from Biodentine. The aim of this study was to evaluate the effects of DR HEDP and EDTA on the calcium ion release and compressive strength of Biodentine. The null hypothesis tested was, there is no influence of DR HEDP, EDTA, and NaOCl on the calcium ion release and compressive strength of Biodentine.

## 2 Materials & methods

Ethical clearance was obtained from the Institutional ethical committee (834/2019); no informed consent was required. For this in vitro study, the sample size was calculated based on the results of the previous study [29] with reference to 95 % confidence level and 90 % power with the mean difference of 11.9 and standard deviation of 8.1 in relation to calcium ion release and compressive strength of TCS cement. The sample size per group was estimated to be 10.

### 2.1 Evaluation of calcium ion release

A single-dose container (0.20 mL) of Biodentine liquid was poured into the Biodentine powder capsule (700 mg) and mixed for 30 s at 4,000–4,200 rpm as per the manufacturer's recommendation. Hand pluggers **(**Dentsply Maillefer, USA) were used to compress Biodentine mix into polyethylene tubes with a 4 mm internal diameter and a 6 mm height. The tubes were weighed to ensure that the volume of cement was consistent in each tube after they were filled. Forty Biodentine cylindrical specimens were moulded and kept for 7 days at 37 °C with 100 % humidity in an incubator [30]. Later, a scalpel was used to separate the set material from the polyethylene tubes. Samples were divided randomly into four groups (n = 10). In group 1, samples were soaked with a 17 % EDTA (Vista Dental Products, Racine, WI, USA) solution with pH of 8.5**.** In group 2, samples were soaked with DR HEDP (Medcem) solution with pH of 11.5**.** DR HEDP (Medcem) is a capsule that contains 0.9 g of etidronate powder, which was mixed with 10 mL of 3 % NaOCl solution (Vista Dental). In group 3, samples were soaked with 3 % NaOCl (Vista Dental) with pH of 12 and in group 4, samples were soaked with distilled water (control) with pH of 7. All samples were individually immersed in falcon tubes containing 10 mL of the test solutions for 1 min, and 10 separate measurements were made. The mean concentration of the calcium ions released after 1 min was measured using atomic absorption spectrophotometry (Thermoscientific, Ice 3000 series, MIT Physics Lab, Karnataka, India) with a calcium-specific hollow cathode lamp. A standard stock solution was diluted with water to create concentrations of 100, 200, 400, 600, and 800 ppm to check the instrument calibration. For each concentration, was a standard curve created based on measurements of the solutions.

### 2.2 Compressive strength evaluation

**Fig. 1:**
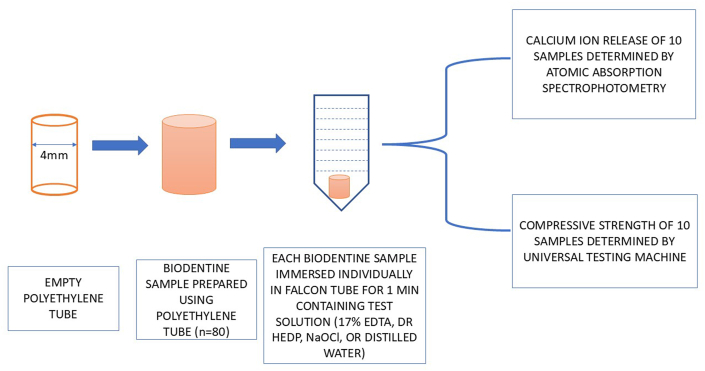
Schematic representation of the methodology employed for the evaluation of calcium ion release and compressive strength of Biodentine.

A single-dose container (0.20 mL) of Biodentine liquid was poured into the Biodentine powder capsule (700 mg) and mixed for 30 s at 4,000–4,200 rpm as per manufacturer's recommendation. Using a plugger, the material was packed into 40 cylindrical polyethylene molds with internal dimensions of 6 mm height and 4 mm width, and the surface was leveled. The assembly was incubated at 37 °C with 100 % humidity for 7 days. Then, 400-grit sandpaper was used to eliminate material flash from the molds' ends. The samples were randomly divided into four groups (n = 10) and treated identically to the method for calcium ion release. CS of specimen was then determined by inserting the samples along the length in between the compression plates of a universal testing machine (Instron model 1011, UK) at a crosshead speed of 1 mm/min. The force required to fracture the samples (in N/mm2) was then according to the ISO 9917-1 determined [31]. The experimental scheme for the evaluation of calcium ion release and CS is represented in Fig. 1.

### 2.3 Statistical analysis

Statistical analysis was performed using SPSS (Statistical Package for the Social Sciences) software (PASW Statistics 18; SPSS Inc., Chicago, IL, USA) version 23.0 with the significance level at α = 0.05. The normality of the data was tested using Kolmogorov-Smirnov test. Group comparisons were performed using One Way ANOVA (Fishers F test); multiple comparisons were done using Tukey Honestly Significant Difference test.

## 3 Results

### 3.1 Calcium ion release

**Fig. 2:**
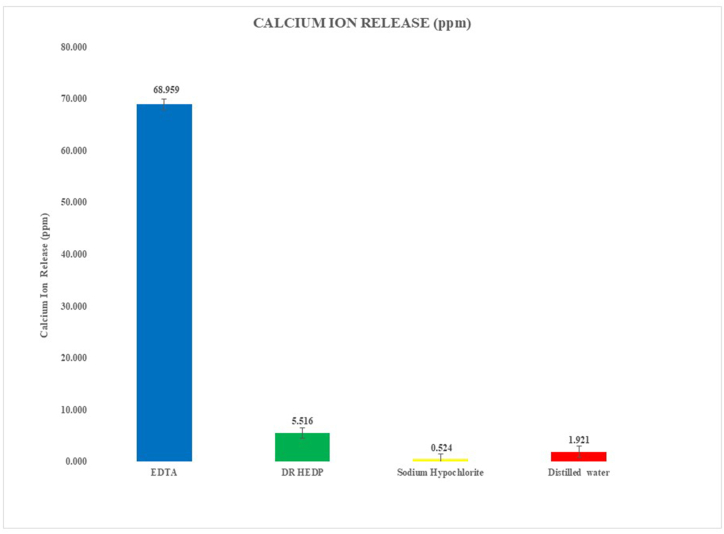
Bar diagram representing calcium ion release from Biodentine after treatment with four experimental solutions.

Fig. 2 and Table 1 show the calcium release results (ppm). The lowest calcium ion release was measured for the 3 % NaOCl (p < 0.001), while samples soaked in 17 % EDTA had the highest release of calcium ions from Biodentine (p < 0.001). On intergroup comparison, no significant differences were determined between 3 % NaOCl and DR HEDP (p = 0.23), distilled water and 3 % NaOCl (p = 0.94), or DR HEDP and distilled water (p = 0.51).

**Table 1:** Calcium ion released (mean ± standard deviation, expressed as ppm) after treatment with four irrigation solutions.

Calcium Ion Release (ppm)				
	Mean	Std. Deviation	Minimum	Maximum
DR HEDP	5.5155	1.5916	3.3016	8.7274
EDTA	68.9587	11.4038	51.0706	87.2998
Distilled water	01.9214	0.6711	0.4746	2.6801
Sodium Hypochlorite	0.5236	0.1225	0.2533	0.7169

F = 331.774, p < 0.001.

### 3.2 Compressive strength

**Fig. 3:**
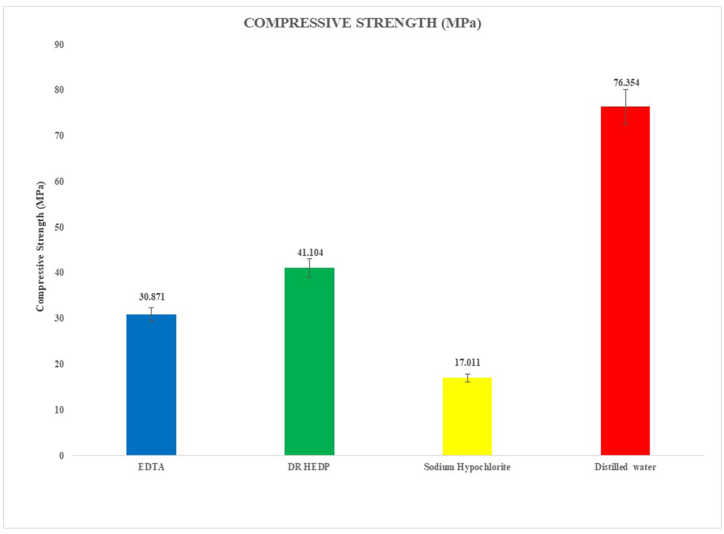
Bar diagram representing compressive strength of Biodentine after treatment with four experimental solutions.

Fig. 3 and Table 2 illustrate the CS values of the experimental results, both means and standard deviations (MPa). The lowest CS values were for samples treated with 3 % NaOCl (p < 0.001), followed by EDTA and then DR HEDP and with distilled water (p < 0.001). When EDTA and DR HEDP were compared, EDTA reduced the CS significantly more (p = 0.019).

**Table 2:** Compressive strength (mean ± standard deviation, expressed as MPa) after treatment with four irrigation solutions.

Comprehensive Strength (MPa)				
	Mean	Std. Deviation	Minimum	Maximum
DR HEDP	41.1040	5.5704	35.4600	50.2500
EDTA	30.8710	7.2359	11.1500	36.7600
Distilled water	76.3540	10.2613	60.8900	86.5700
Sodium Hypochlorite	17.0110	1.5369	13.7700	18.6700

F = 134.528, p < 0.001.

## 4 Discussion

This study evaluated the effect of root canal irrigating solutions: DR HEDP, 17 % EDTA, and 3 % NaOCl, on the calcium ion release and CS of Biodentine. Significant differences were determined among the irrigating solutions for calcium ion release and CS; the null hypothesis was rejected.

In the present study, 3 % NaOCl was used, which has similar clinical outcome to higher concentrations [32]. Distilled water, which was used as a control, caused some calcium ion release. Clinically chelators are used as a final irrigant for 1 min, which is why this time was chosen [17]. The 17 % EDTA solution caused the most ion release. This is in agreement with Bayram et al. [30] who reported that TCS-based materials (Bioaggregate, white MTA, and Biodentine) when treated with EDTA, showed the highest release of calcium ions. The presence of calcium chloride in Biodentine fluid and the cement's fast setting/hydration can be linked to its increased calcium ion release [30]. EDTA is a strong chelator (pH of 8.5) and HEDP is a mild chelator (pH of 11.5), which explains the higher Ca ion release of EDTA. However, Neelakantan et al. [33] reported the highest Ca ion release from another TCS cement (MTA Plus) with saline when compared to NaOCl, EDTA, mixture of NaOCl and etidronic acid, or QMix.

Biodentine when used as a reparative or sealing material needs high CS for use as a temporary restorative. Grech et al. [34] reported that Biodentine has the highest CS compared to the other materials evaluated in their study. Kayahan et al. [35] examined CS to see if there were any changes in CS after etching. After 7 days, they found that acid etching after 7 days of setting did not affect the CS of ProRoot MTA or Biodentine [35]. The CS of cement is important [36]. For perforation repair or apexification, the irrigation solution may directly contact with Biodentine and can reduce its CS. In the present study, Biodentine samples treated with NaOCl had lowest CS but Govindaraju et al. [29], found that the CS of cement was not significantly reduced by NaOCl, unlike in the present study. This difference in these results may be attributed to the design of the study, and volume of NaOCl used. In the present study, the Biodentine samples were completely immersed in 10 mL of NaOCl. However, in previous study [29] samples were treated superficially with saturated moistened gauze containing 3 mL of NaOCl. The DR HEDP, used in conjunction with 3 % NaOCl reduced the CS of Biodentine comparatively less to EDTA. Hence, DR HEDP can be used to avoid the attack of EDTA on Biodentine matrix. The detrimental effect of EDTA on Ca ion release and CS of Biodentine may also be attributed to its pH, which is less alkaline when compared to DR HEDP. Previous studies have demonstrated that acidic pH can affect the physical and chemical properties of TSC cements [37,38]. In the present study, since NaOCl had detrimental effect on CS of Biodentine, a final rinse with distilled water after the use of NaOCl is advisable. Results of the present study have a few limitations and may not necessarily be extrapolated to the clinical scenario. Firstly, only one type of TCS cement (Biodentine) was tested. Future studies should evaluate the effect of EDTA, Dual Rinse HEDP and NaOCl on various other TCS based cements used in endodontic treatment. Secondly, the Biodentine samples were completely immersed into the irrigating solutions. However, this approach does not accurately replicate the clinical endodontic setting. In real-life root canal treatment, irrigants are introduced into root canals via irrigation needles with minimal contact of the irrigating solutions with the TCS materials which are used after irrigation for repair or sealing. Hence, the conclusions drawn from the present study may overestimate the detrimental effects of irrigating solutions on Biodentine.

## 5 Conclusion

The 17 % EDTA solution dissolved more calcium ions from Biodentine than DR HEDP. NaOCl released the fewest calcium ions. The compressive strength of Biodentine was higher when treated with DR HEDP when compared to 17 % EDTA. NaOCl reduced the compressive strength of Biodentine compared to the control of distilled water.

## Data availability statement

Data will be made available on request.

## CRediT authorship contribution statement

**Devika Jain:** Writing – original draft, Formal analysis, Data curation, Conceptualization. **Krishna Prasad Shetty:** Writing – review & editing, Formal analysis, Conceptualization. **Alexander Maniangat Luke:** Writing – review & editing, Formal analysis. **Nidambur Vasudev Ballal:** Writing – review & editing, Writing – original draft, Supervision, Methodology, Formal analysis, Data curation, Conceptualization.

## Declaration of competing interest

The authors declare that they have no known competing financial interests or personal relationships that could have appeared to influence the work reported in this paper.
